# Isoleucyl-tRNA synthetase 2 promotes pancreatic ductal adenocarcinoma proliferation and metastasis by stabilizing β-catenin

**DOI:** 10.1016/j.gendis.2024.101382

**Published:** 2024-07-24

**Authors:** Yixun Jin, Xinyang Huang, Zhuoxin Wang, Berik Kouken, Qi Wang, Lifu Wang

**Affiliations:** Department of Gastroenterology, Ruijin Hospital, Shanghai Jiao Tong University School of Medicine, Shanghai 200001, China

**Keywords:** Aminoacyl-tRNA synthetases, Isoleucyl-tRNA synthetase 2, Pancreatic ductal adenocarcinoma, WNT signaling pathway, β-Catenin

## Abstract

Isoleucyl-tRNA synthetase 2 (IARS2), originally regarded as an enzyme ligating isoleucine to the corresponding tRNA, has been identified as an oncogene recently. However, its function in pancreatic ductal adenocarcinoma (PDAC) remains to be discovered. Here we explored the biological role of IARS2 in PDAC. Up-regulated IARS2 was found in PDAC tissues and cell lines. Kaplan–Meier survival analysis indicated a worse prognosis in patients with high IARS2 expression. CCK-8, EdU, and colony formation assays showed IARS2 overexpression enhanced PDAC proliferation, which was reduced by IARS2 knockdown. Meanwhile, IARS2 down-regulation inhibited PDAC metastasis by impeding epithelial–mesenchymal transition. These results were also supported by subcutaneous xenograft and metastasis assays *in vivo*. To figure out underlying mechanisms, differential and enrichment analyses were conducted and the WNT signaling pathway was discovered. Our results demonstrated that there was no significant relationship between the WNT signaling pathway key factor CTNNB1 and IARS2 at the transcription level. However, cycloheximide assays showed that IARS2 reduced the β-catenin degradation rate. IARS2 inhibited the phosphorylation of β-catenin at the Ser33/37 site and regulated downstream targets of WNT signaling including c-MYC, c-JUN, and MMP7. The enhancement of proliferation and metastasis caused by IARS2 could be reversed by MSAB, an agent that promotes β-catenin degradation. In summary, IARS2 facilitates PDAC proliferation and metastasis by stabilizing β-catenin, which leads to WNT/β-catenin activation. IARS2 serves as an underlying prognosis marker and a potential therapeutic target for PDAC.

## Introduction

With the lowest 5-year relative survival rate in cancer, pancreatic ductal adenocarcinoma (PDAC) has become the fourth leading cause of cancer-related death.^1^ However, the effect of therapeutic modalities on PDAC is far from satisfactory. Further molecular taxonomy of PDAC phenotypes may provide diverse avenues for therapeutic intervention.^2^

Currently, advances in genomics and transcriptomics revealed a subset of PDAC with WNT dependency.^3^ Exhibiting increased WNT/β-catenin transcriptional activity, the WNT-addicted subset of PDAC is sensitive to WNT signaling pathway inhibitors in preclinical studies.^4^, ^5^, ^6^ Accordingly, many therapeutic agents have been developed but most of them have not entered into clinical trials and the registered clinical trials rarely present satisfactory results.^7^, ^8^, ^9^ These results indicate there is other intrinsic or acquired resistance to WNT inhibitors and more in-depth basic research is needed.

Aminoacyl-tRNA synthetases (ARSs), an ancient enzyme family that catalyze the ligation of amino acids to their corresponding tRNAs in protein synthesis, have gained increasing attention for their regulatory function independence of translation.^10^ Bioinformatic analyses and proteomic experiments have demonstrated that ARSs star as a sensor of stimuli and stress in various cancers.^11^ For instance, leucyl-tRNA synthetase is involved in amino acid-induced mTORC1 (mechanistic target of rapamycin complex 1) activation by sensing intracellular leucine concentration^12^ and cysteinyl-tRNA synthetase activates AMPK (AMP-activated protein kinase) under cysteine deprivation conditions for cell survival.^13^ However, there is no evidence linking ARSs with WNT/β-catenin signaling in pancreatic cancer.

Isoleucyl-tRNA synthetase 2 (IARS2), a member of the ARS family, is dysregulated in gastric cancer, non-small cell lung cancer, leukemia, and osteosarcoma.^14^, ^15^, ^16^, ^17^ In this study, we found that up-regulated IARS2 in PDAC tissues was correlated with poor prognosis and unraveled IARS2 functions and mechanisms in PDAC. IARS2 facilitated PDAC proliferation and metastasis by stabilizing β-catenin and activating the WNT/β-catenin pathway. Our results indicate that IARS2 serves as an underlying prognosis marker and a potential therapeutic target for PDAC.

## Materials and methods

### Download of public data

IARS2 transcription level in different cancer types including PDAC was obtained from GEPIA2 (http://gepia2.cancer-pku.cn) website. RNA sequencing data and corresponding clinical information of the TCGA-PAAD cohort were downloaded from the TCGA database (https://portal.gdc.cancer.gov/). IARS2 alteration in multiple cancers was obtained from cBioPortal (https://www.cbioportal.org/).

### Cell culture, reagents, and inhibitors

Human pancreatic cancer cell lines MIA PaCa-2, BxPC-3, PANC-1, and SW1990 were purchased from the Chinese Academy of Sciences Cell Bank. Capan-1 and the normal pancreatic ductal cell line of human hTERT-HPNE were purchased from the American Type Culture Collection. The complete medium was used for cell culture, which was prepared by Dulbecco's modified Eagle's medium (DMEM; cat. no. MA0110; Meilunbio) containing 10% fetal bovine serum and 1% penicillin/streptomycin (cat. no. MA2013; Meilunbio). Cells were cultured at 37 °C with 5% CO_2_. Cycloheximide (CHX), MG-132, and MSAB were purchased from MedChemExpress (cat. no. HY-12320, HY-13259, and HY-120697, respectively; MCE).

### RNA isolation, cDNA synthesis, and protein extraction

TRIzol® reagent (cat. no. 15596026; Invitrogen) was used for total RNA extraction and the quality of extracted RNA was assessed according to the ratio of A260/A280. The procedure was described in the previous literature.^18^ cDNA was synthesized with the First Strand cDNA Synthesis SuperMix (cat. no. 11137ES60; Yeasen) following the instructions. For protein extraction, cells were lysed by RIPA (cat. no. MA0151; Meilunbio), which contained protein protease inhibitors and phosphatase inhibitors. After ultracentrifugation at 4 °C, the supernatant was boiled with 5× SDS-PAGE Protein Loading Buffer (cat. no. 20315ES20; Yeasen) and stored at −20 °C.

### Quantitative real-time PCR (qRT-PCR) and immunoblotting

The procedure of qRT-PCR and immunoblotting were described in the previous literature.^19^ Primer sequences for qRT-PCR and primary antibodies we used are listed in Tables S1 and S2.

### Lentivirus-mediated stable transfection

Interference and overexpression lentiviruses of IARS2 were constructed by GeneChem Company with GV493 and GV705 vectors respectively. Transfection was performed according to the manufacturer's protocol. Following transfection, cells were further treated with 10 μg/mL puromycin to establish stably transfected cells (shIARS2, shCtrl, IARS2, and vector).

### Cell counting kit 8 (CCK-8) assays

For CCK-8 assays, transfected cells (1 × 10^3^ per well) were seeded in 96-well plates. After culturing for 24, 48, 72, 96, and 120 h, 100 μL DMEM containing 10 μL CCK-8 reagent (cat. no. MA0218; Meilunbio) was added to cells. The absorption at 450 nm was recorded following incubation in the dark at 37 °C for 2 h.

### 5-ethynyl-20-deoxyuridine (EdU) assays

For EdU assays, cells (2 × 10^4^ per well) were plated in 24-well plates and cultured overnight. Then the cells were co-cultured with 10 μM EdU (cat. no. C0075S; Beyotime) for 4 h. Rinsed by phosphate buffer saline solution (PBS) twice, cells were fixed with 4% paraformaldehyde (cat. no. G1101-500ML; Servicebio) for 15 min. Incubated with 0.3% TritonX-100 after PBS washing, cells were dyed with reaction solution and observed by fluorescence microscopy.

### Colony formation assays

For colony formation assays, transfected cells were seeded in 6-well plates (5 × 10^2^ per well) and cultured for 2–3 weeks until colonies were visible to the naked eye. Then the cells were incubated with 4% paraformaldehyde for 30 min and stained with crystal violet solution (cat. no. G1014-50ML; Servicebio) for another 15 min at room temperature. The stained colonies were counted manually to assess cell colony formation ability.

### Transwell assays

To evaluate cell migration and invasion capabilities after transfection, transwell assays were conducted. For migration assays, 100 μL DMEM containing 5 × 10^4^ cells was added to the upper transwell chambers and 600 μL complete medium was added to the lower chambers. Following 24 h incubation, migrated cells were fixed using 4% paraformaldehyde. Cells in the upper chambers were removed softly by cotton-tipped swabs after crystal violet staining. Finally, the cells were observed under a microscope. Invasion assays were performed following the same procedure except the upper chambers were pre-coated with Matrigel (cat. no. 356234; Corning) and cell incubation time was extended to 48 h.

### Subcutaneous xenograft and metastasis assay *in vivo*

Briefly, BxPC-3 cells and PANC-1 cells stably transfected with empty vector, IARS2, shCtrl, or shIARS2 were washed three times by PBS and finally suspended in 100 μL PBS. For subcutaneous xenograft assay, 3 × 10^6^ cells were subcutaneously injected into the right flank of 4-week-old female BALB/c nude mice (*n* = 5/group). Tumor size was measured each other day and the volume was calculated according to the following equation: tumor volume = length × width^2^/2. BxPC-3 tumor-bearing mice were sacrificed after 2 weeks and PANC-1 tumor-bearing mice were sacrificed after 6 weeks, and then the subcutaneous tumor was removed, measured, and weighed. Ki-67 expression level in subcutaneous tumors was evaluated by immunohistochemical tests.

For metastasis analysis, 1 × 10^6^ transfected BxPC-3 cells were injected into the tail vein and the mice were euthanized after 5 weeks. Following formalin-fixed and paraffin-embedded, slices of lung tissue were stained with hematoxylin–eosin and the metastasis nodules were calculated under a microscope. All animal experiments were approved by the Institutional Animal Care and Use Committee of Shanghai Jiao Tong University (IACUC approval no. A-2023-097).

### Identification of differentially expressed genes and analysis of enrichment and immune-cell infiltration

Differentially expressed genes between the IARS2 high expression group and low expression group were identified by “DESeq2” R packages. Kyoto Encyclopedia of Genes and Genomes (KEGG) pathway analyses of common differentially expressed genes were carried out by the “clusterProfiler” package. Gene set enrichment analysis (GSEA) was performed by GSEA_4.1.0.

To analyze immune cell infiltration in PDAC samples from TCGA, we used the LM22 gene signature, which contains 547 genes and discriminates 22 human hematopoietic cell phenotypes, to calculate different immune cell proportions. Deconvolution algorithm CIBERSORT and single sample gene set enrichment analysis (ssGSEA) were applied to estimate the immune cell infiltrations.^20^ All these analyses were conducted by R (4.1.0 version) software.

### CHX chase assay and MG-132 assay

Briefly, transfected pancreatic cancer cells were seeded in 24-well plates and cultured overnight. Then cells were treated with CHX (100 μg/mL) or MG-132 (10 μM) and harvested at the timepoints of interest. Cell lysates were analyzed by immunoblotting with anti-β-catenin, anti-IARS2, or anti-β-actin antibodies.

### Statistical analysis

All data in this study were analyzed with GraphPad Prism 9 and R (4.1.0 version) software. Student's *t*-test was used for comparison of two independent groups. One-way ANOVA followed by the least significant difference post hoc test was used for evaluating statistical significance among multiple groups. Survival analysis was performed using the Kaplan–Meier method and log-rank test. *P* < 0.05 was defined as statistically significant.

## Results

### IARS2 highly expressed in PDAC tissue and cell lines

**Figure 1:**
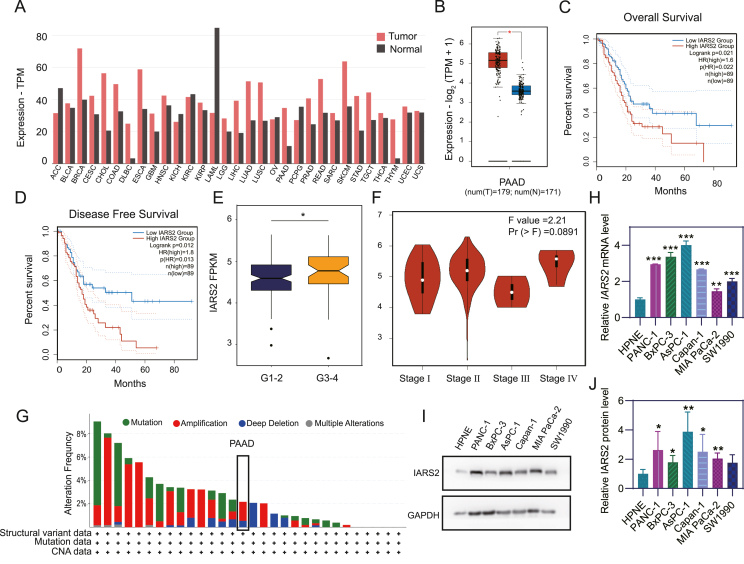
IARS2 was up-regulated in PDAC tissue and cell lines. **(A)** Bar plot of IARS2 transcription level in different cancer types. **(B)** TCGA and GTEx databases showed IARS2 transcription level was higher in tumor tissue compared with normal pancreas tissue. **(C)** High IARS2 expression linked to shorter overall survival time. **(D)** Disease free survival time was shortened in high IARS2 expression group. **(E)** IARS2 transcription level was elevated in grade 3–4 PDAC compared with grade 1–2 PDAC. **(F)** No difference of IARS2 transcription level was detected among pancreatic cancer at different stages. **(G)** Alteration of IARS2 in TCGA pancreatic adenocarcinoma cohort. **(H)** IARS2 mRNA transcription was up-regulated in pancreatic adenocarcinoma cell lines compared with normal cell lines. **(I)** IARS2 protein expression was elevated in pancreatic adenocarcinoma cell lines. **(J)** Statistical analysis of IARS2 western blotting results. Grey value of brands was measured by ImageJ and data represented the mean ± standard deviation of three experiments. ∗*P* < 0.05, ∗∗*P* < 0.01, ∗∗∗*P* < 0.001. IARS2, isoleucyl-tRNA synthetase 2; PDAC, pancreatic ductal adenocarcinoma.

We analyzed IARS2 transcription pattern in different cancer types via GEPIA2 and detected higher expression in cholangiocarcinoma, lymphoid neoplasm diffuse large B-cell lymphoma, pancreatic adenocarcinoma, and thymoma as well as lower expression in acute myeloid leukemia compared with normal tissue (Fig. 1A, B). Kaplan–Meier curves showed pancreatic cancer patients with higher IARS2 expression had shorter overall survival time (*P* = 0.02; Fig. 1C) and disease-free survival time (*P* = 0.01; Fig. 1D). Irrelevant to patient gender or age, IARS2 transcription level was significantly higher in pancreatic cancer at tumor grade 3–4 compared with grade 1–2 (*P* = 0.02; Fig. 1E; Fig. S1). However, the expression level of IARS2 did not reflect the progression of TNM staging, as no difference was shown between stage Ⅰ and stage Ⅳ in the TCGA-PAAD cohort (Fig. 1F). The total alteration rate of IARS2 in TCGA-PAAD cohort is 3%, the major of which is amplification alteration (Fig. 1G). Next, IARS2 expression level was evaluated in one normal (hTERT-HPNE) and six PDAC (BxPC-3, SW1990, MIA PaCa-2, Capan-1, PANC-1, and AsPC-1) cell lines by qRT-PCR and immunoblotting. mRNA transcription level was increased in six PDAC cell lines, which varied from 1.5-fold to 4-fold compared with hTERT-HPNE (Fig. 1H). Western blotting showed that IARS2 was significantly up-regulated in five PDAC cell lines, with maximum amounts in PANC-1 and AsPC-1 cells (Fig. 1I, J).

### IARS2 facilitated PDAC proliferation *in vitro*

**Figure 2:**
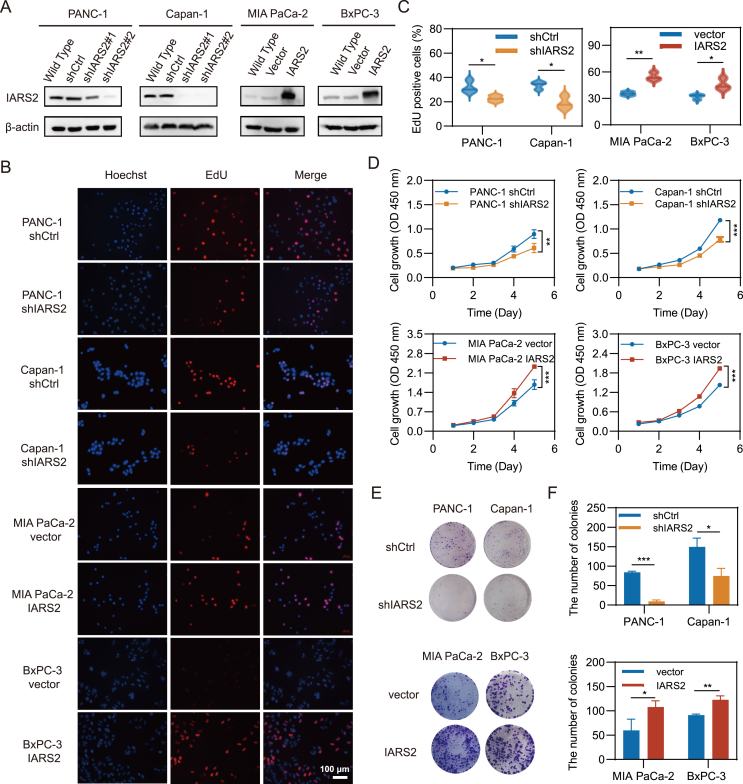
IARS2 promoted PDAC proliferation *in vitro*. **(A)** The protein level of IARS2 in PANC-1 and Capan-1 cells after transfected with shIARS2 or shCtrl, and in MIA PaCa-2 and BxPC-3 cells transfected with empty vector (vector) or lentivirus-mediated flag-tagged overexpression IARS2 (IARS2). **(B)** The proliferation of PANC-1 and Capan-1 cells following knockdown of IARS2 and the proliferation of MIA-PaCa-2 and BxPC-3 cells after IARS2 overexpression showed by Edu assays. **(C)** Statistical analysis of EdU assays. **(D)** CCK-8 assays demonstrated cell proliferation was decreased by IARS2 knockdown and increased by IARS2 overexpression. **(E)** IARS2 knockdown inhibited colony formation of PDAC cells, but the overexpression of IARS2 increased colony formation. **(F)** Statistical analysis of colony formation assays. Data represented the mean ± standard deviation of at least three experiments. ∗*P* < 0.05, ∗∗*P* < 0.01, ∗∗∗*P* < 0.001. IARS2, isoleucyl-tRNA synthetase 2; PDAC, pancreatic ductal adenocarcinoma.

Since a higher IARS2 expression level was correlated with a worse prognosis, we hypothesized that IARS2 might promote PDAC growth. According to IARS2 expression level in different cell lines, PANC-1 and Capan-1 cells were selected to construct IARS2 knockdown cell lines, while MIA PaCa-2 and BxPC-3 cells for overexpression (Fig. 2A). EdU assays showed IARS2 depletion reduced DNA synthesis, as EdU positive staining percentage of PANC-1 and Capan-1 cells dropped from 32.05% to 22.53% (*P* = 0.04) and 33.63% to 19.01% (*P* = 0.02) respectively. Conversely, IARS2 overexpression enhanced DNA synthesis, as EdU positive staining percentage of MIA PaCa-2 and BxPC-3 cells increased from 35.30% to 54.42% (*P* < 0.01) and 31.87% to 45.63% (*P* = 0.03), respectively (Fig. 2B and C). The impact of IARS2 knockdown on cell proliferation was further measured by the CCK-8 assays, which presented significant growth inhibition compared with vehicle-treated cells (*P* < 0.01). Overexpression of IARS2 exerted opposite effects in MIA PaCa-2 and BxPC-3 (Fig. 2D). Additionally, IARS2 knockdown impeded while overexpression enhanced colony formation ability of PDAC cells (*P* < 0.05; Fig. 2E, F). These findings suggested that IARS2 promoted PDAC proliferation *in vitro*.

### IARS2 promoted PDAC migration and invasion via EMT

**Figure 3:**
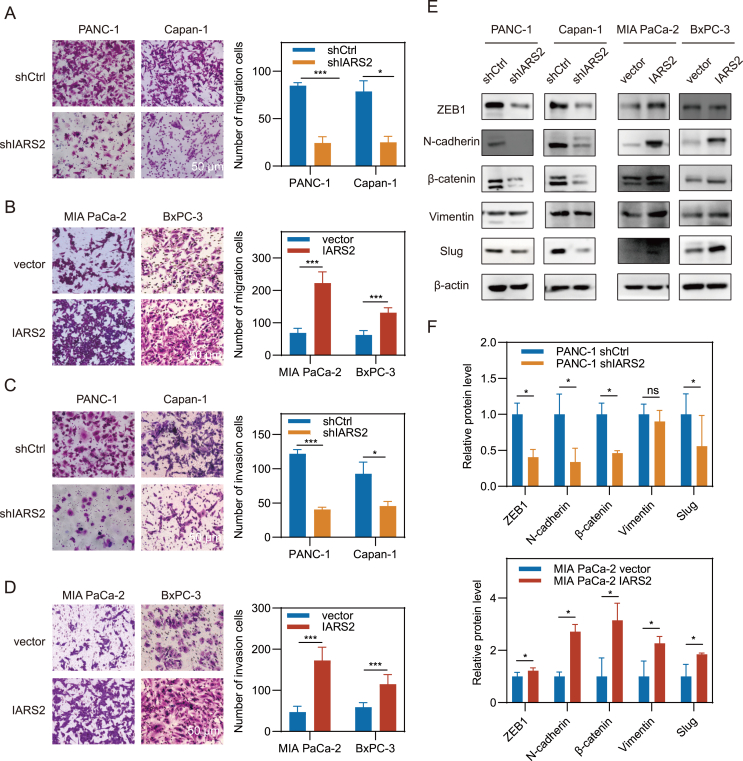
IARS2 enhanced PDAC migration and invasion via EMT. **(A**–**D)** Transwell migration and Matrigel invasion assays showed that knockdown of IARS2 reduced migration and invasion cells while overexpression of IARS2 increased migration and invasion cells. **(E)** The expression levels of EMT related protein in modified PDAC cell lines were assayed by western blotting. β-Actin was used as an internal control. **(F)** Statistical analyses of EMT related protein expression level in PANC-1 and MIA PaCa-2 cell lines. Grey value of brands was measured by ImageJ and data represented the mean ± standard deviation of at least three experiments. ∗*P* < 0.05, ∗∗*P* < 0.01, ∗∗∗*P* < 0.001. IARS2, isoleucyl-tRNA synthetase 2; PDAC, pancreatic ductal adenocarcinoma; EMT, epithelial–mesenchymal transition.

As highly expressed IARS2 was linked to shorter disease-free time, IARS2 was probably involved in PDAC metastasis and recurrence. Transwell assays were conducted to explore the impact of IARS2 on the PDAC malignant phenotype *in vitro*. Results demonstrated that IARS2 deficiency significantly hindered cell migration and invasion in PANC-1 and Capan-1 cells, but its overexpression strikingly promoted migration and invasion in MIA PaCa-2 and BxPC-3 cells (*P* < 0.05; Fig. 3A–D). Considering that epithelial–mesenchymal transition (EMT) is a key event in PDAC metastasis, we assessed the expression level of EMT markers and regulatory transcription factors by immunoblotting. IARS2 knockdown down-regulated N-cadherin, ZEB1 (Zinc finger E-box binding homeobox 1), β-catenin, and Slug at the protein level, while overexpression showed opposite results, indicating that IARS2 may facilitate EMT via up-regulation of transcription factors (Fig. 3E, F; Fig. S2).

### IARS2 enhanced PDAC proliferation and metastasis *in vivo*

**Figure 4:**
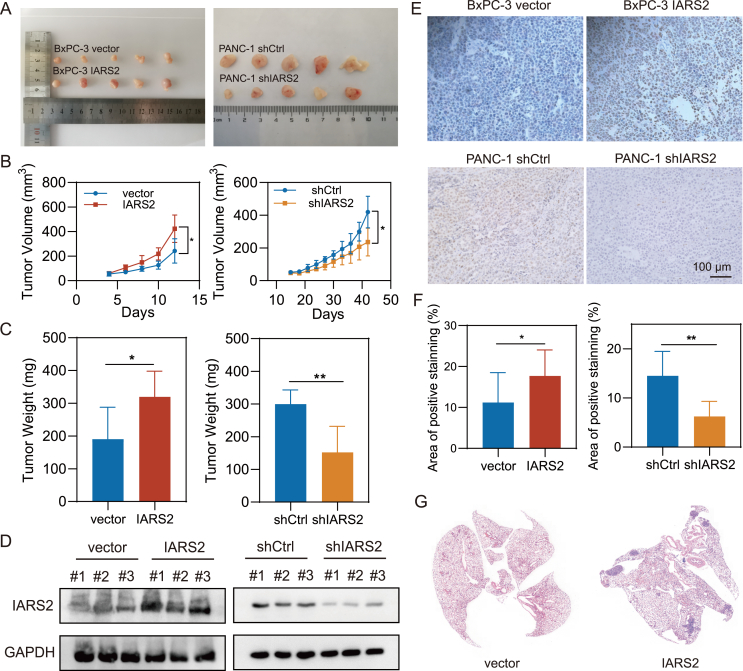
IARS2 enhanced PDAC proliferation and metastasis *in vivo*. **(A)** Images of subcutaneous tumor removed from mice (*n* = 5 per group). **(B)** Growth curves showed that overexpression of IARS2 promoted tumor growth while IARS2 knockdown exerted the opposite effect in nude mice. **(C)** Tumor weight was increased in the IARS2 group and decreased in the shIARS2 group. **(D)** The protein level of IARS2 in tumor tissues. GAPDH was used as an internal control. **(E)** Ki-67 immunohistochemical images of subcutaneous tumor in different groups. **(F)** Statistical analysis showed that IARS2 overexpression increased positive Ki-67 staining cells and IARS2 knockdown reduced positive Ki-67 staining cells in tumor tissues. **(G)** Hematoxylin–eosin staining of nude mice lung after injection of BxPC-3 transfected with vector or IARS2 overexpression lentivirus. ∗*P* < 0.05, ∗∗*P* < 0.01, ∗∗∗*P* < 0.001. IARS2, isoleucyl-tRNA synthetase 2; PDAC, pancreatic ductal adenocarcinoma.

Next, we investigated the *in vivo* effect of IARS2 in tumor proliferation by subcutaneous xenograft assays in nude mice (Fig. 4A). Tumor size was measured each other day and the growth curve showed IARS2 overexpression boosted tumor growth (*P* = 0.03), while knockdown exhibited opposite effect (*P* = 0.01; Fig. 4B; Fig. S3). Compared with the average tumor weight of 190.6 mg in BxPC-3 vector group, tumor weight was significantly higher in BxPC-3 IARS2 group, which was 319.8 mg (*P* = 0.04). Conversely, the average tumor weight of the PANC-1 shIARS2 group was 152.2 mg, while the average tumor weight was 299.6 mg in the PANC-1 shCtrl group, which was nearly twice that of the shIARS2 group (*P* < 0.01; Fig. 4C). These results indicated that high IARS2 expression significantly increased tumor size and weight. Subsequently, IARS2 expression in subcutaneous tumors of these groups was validated by immunoblotting (*P* < 0.05; Fig. 4D; Fig. S4). Subcutaneous tumor tissues were stained with Ki-67 antibody and the representative images were displayed in Figure 4E. Consistent with the *in vitro* findings, Ki-67 staining detected a higher percentage of proliferating cells in high IARS2 expression xenografts (*P* < 0.05; Fig. 4F).

In addition, tail vein metastasis assays were used to validate that IARS2 could promote PDAC metastasis *in vivo*, and the result was consistent with the *in vitro* results. BxPC-3 cells transfected with empty vector or lentivirus overexpressed IARS2 were injected into the tail vein and histological examination of the lung was performed after 5 weeks. Hematoxylin–eosin staining showed that all mice (5/5) bearing IARS2 overexpression cells presented lung metastases, while 60% (3/5) of the mice in the vector group showed lung metastasis (Fig. S5). Representative pictures were shown in Fig. 4G. Furthermore, metastatic nodules were significantly increased in the IARS2 group compared with the vector group (*P* = 0.01; Fig. S6). These results demonstrate that IARS2 is a key factor in PDAC proliferation and metastasis.

### Transcriptomics analysis revealed IARS2 involvement in WNT signaling and cancer cell stemness

**Figure 5:**
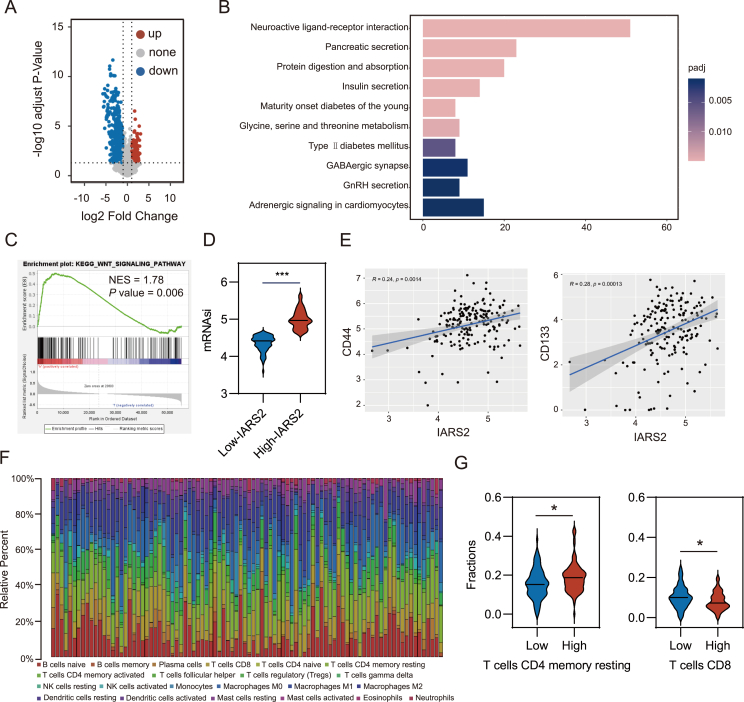
Transcriptomics analysis revealed IARS2-induced biological process. **(A)** Volcano plot of the quantitative gene expression from TCGA-PAAD patients with high IARS2 expression and low IARS2 expression. Genes differentially expressed with fold change over 1 were marked in blue and red. **(B)** The top enriched signaling pathway based on KEGG analysis with the RNA sequencing data of TCGA-PAAD. **(C)** GSEA showed the enrichment pathway in high-IARS2 expression group of TCGA-PAAD. **(D)** The patients with high IARS2 expression possessed higher mRNAsi score. **(E)** IARS2 was positively associated with stemness markers including CD44 and CD133. **(F)** Bar charts of 22 immune cell proportions in 179 PDAC tissues from TCGA databases. **(G)** Violin plot of memory resting CD4 T cell and CD8 T cell infiltration in the low and high IARS2 expression groups. ∗*P* < 0.05, ∗∗*P* < 0.01, ∗∗∗*P* < 0.001. IARS2, isoleucyl-tRNA synthetase 2; PDAC, pancreatic ductal adenocarcinoma; CD44, cluster of differentiation 44; CD133, cluster of differentiation 133.

To figure out underlying mechanisms, the TCGA-PAAD cohort was used for differential analysis. A total of 1576 differentially expressed genes were obtained with a cut-off fold change of 1 and *P* < 0.05. Of these, 230 genes were up-regulated and 1346 genes were down-regulated (Fig. 5A). KEGG enrichment analysis presented a distribution of differentially expressed genes in pathways relating to pancreatic secretion, diabetes, and amino acid metabolism (Fig. 5B). GSEA analysis showed WNT signaling, cell cycle, p53, and apoptosis pathway were significantly enriched (Fig. 5C; Fig. S7).

Involved in multiple cellular activities, WNT signaling pathway was activated at an early stage of PDAC development and played an important role in stemness maintenance of cancer cells, which prompted us to explore the correlation between IARS2 and stemness.^21^^,^^22^ Gene expression-based stemness index (mRNAsi), which was developed by Tathiane M Malta et al. was used to reflect tumor stemness.^23^ Compared with patients with low IARS2 expression, mRNAsi was higher in patients with high IARS2 expression (*P* < 0.01; Fig. 4D). Spearman correlation analysis showed that expression level of stemness markers including CD44 (cluster of differentiation 44), MET (MET proto-oncogene receptor tyrosine kinase), CD133 (cluster of differentiation 133), FUT4 (fucosyltransferase 4), ACVR1 (activin A receptor type 1), mTOR (mechanistic target of rapamycin kinase), and KLF4 (Krüppel-like factor 4) were positively correlated with IARS2 level, indicating the regulation of cancer cell stemness might be a reason for IARS2 promoting metastasis (Fig. 4E; Fig. S8).

Additionally, we evaluated 22 immune cell proportions in 179 PDAC samples from TCGA-PAAD (Fig. 5F) and compared infiltration levels between the high- and low-IARS2 group based on the CIBERSORT algorithm (Fig. S9). Results revealed that the infiltration level of memory resting CD4 T cell was higher while the CD8 T cell infiltration level was lower in the high-IARS2 group in contrast to the low-IARS2 group (Fig. 5G). Considering that CD8 T cells were responsible for anti-tumor responses, we speculated that high IARS2 expression could correlate with immunosuppressive microenvironment. To validate the impact that IARS2 exerted on immunity, we re-analyzed the immune cell infiltration level using ssGSEA and obtained similar results (Fig. S10), which supported our speculation.

### IARS2 regulated WNT signaling by inhibition of β-catenin degradation

**Figure 6:**
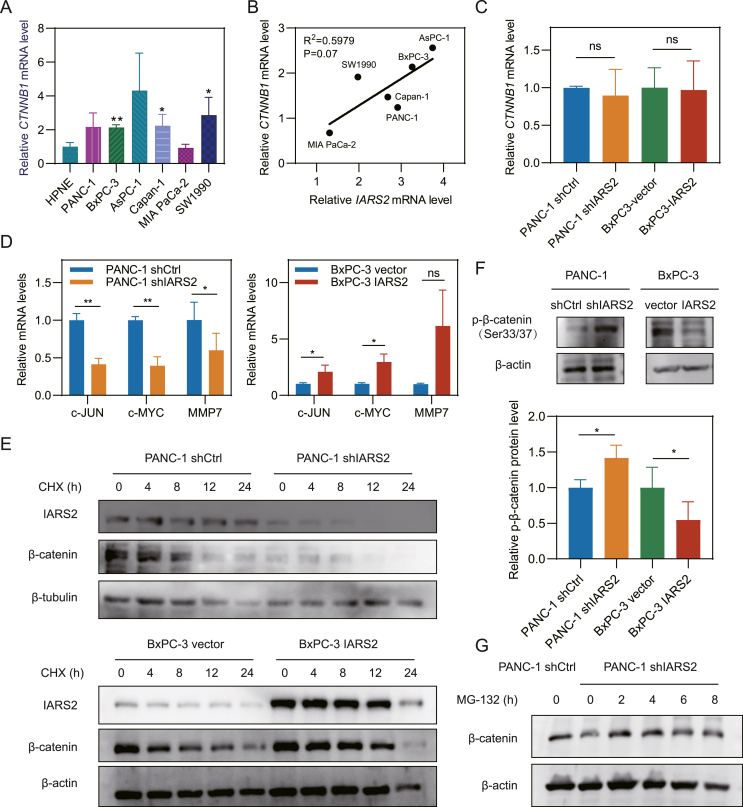
IARS2 affected WNT signaling via inhibiting degradation of β-catenin. **(A)** Relative CTNNB1 mRNA level in normal and pancreatic adenocarcinoma cell lines. **(B)** The correlation between relative IARS2 mRNA transcription level and CTNNB1 mRNA transcription level. **(C)** CTNNB1 mRNA level remained unchanged after IARS2 knockdown or overexpression. **(D)** Downstream targets of CTNNB1 were up-regulated in IARS2 overexpressed cell line and down-regulated in IARS2 knocked down cell line. **(E)** Cycloheximide assays showed IARS2 regulated the protein half-life of IARS2. **(F)** IARS2 knockdown increased β-catenin phosphorylation at Ser33/37 site while overexpression reduced phosphorylation. **(G)** Proteasome inhibitor MG-132 (10 μM) restored β-catenin expression in IARS2 silenced PANC-1 cells. Grey value of brands was measured by ImageJ and data represented the mean ± standard deviation of at least three experiments. ∗*P* < 0.05, ∗∗*P* < 0.01, ∗∗∗*P* < 0.001. IARS2, isoleucyl-tRNA synthetase 2; CTNNB1, catenin beta 1.

Inspired by the GSEA result that WNT signaling was enriched in the high IARS2 expression group and IARS2 knockdown reduced β-catenin expression level, we hypothesized that IARS2 might regulate WNT signaling via β-catenin. First CTNNB1 (catenin beta 1) transcription level was evaluated in hTERT-HPNE and six PDAC cell lines (Fig. 6A). There was no significant correlation between the transcription level of IARS2 and CTNNB1 (Fig. 6B). Meanwhile, CTNNB1 was not altered by IARS2 knockdown or overexpression in PANC-1 or BxPC-3 cell lines respectively (Fig. 6C). However, c-MYC, c-Jun, and MMP7 (matrix metallopeptidase 7), which are downstream genes of β-catenin/TCF4, were down-regulated in IARS2 knocked down PANC-1 cells while up-regulated in IARS2 overexpressed BxPC-3 cells, validating the impact of IARS2 on WNT signaling (Fig. 6D). These results hinted that IARS2 might up-regulate β-catenin by inhibiting degradation instead of promoting transcription. CHX chase assay demonstrated that IARS2 overexpression slowed down the degradation of β-catenin, while IARS2 knockdown accelerated this process (Fig. 6E). Phosphorylation of β-catenin at Ser33/37 site was decreased in IARS2 knocked down PANC-1 cells and increased in IARS2 overexpressed BxPC-3 cells, showing that IARS2 could protect β-catenin from phosphorylation-dependent proteasome degradation by β-TrCP (F-box protein beta-transducin repeat containing protein) (Fig. 6F). Proteasome inhibitor MG-132 reversed β-catenin decrease in PANC-1 cells after IARS2 knockdown (Fig. 6G).

### Promotion of β-catenin degradation impeded IARS2 tumorigenesis function

**Figure 7:**
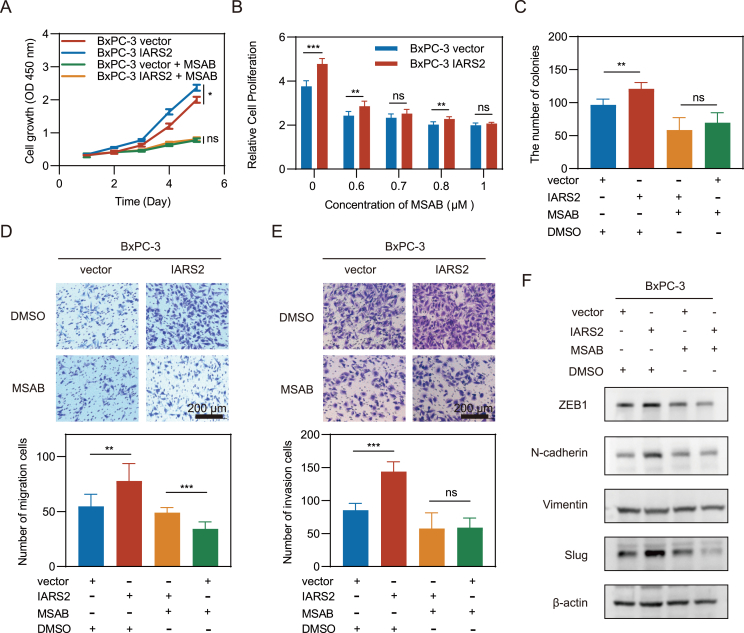
Promotion of β-catenin degradation impeded IARS2 tumorigenesis function. **(A)** Proliferation curve of pancreatic cancer cells following treatment of 1 μM MSAB. **(B)** Relative proliferation rate after treatment with different concentrations of MASB for 96 h. **(C)** MSAB reversed colony formation of IARS2 overexpressed BxPC-3 cells. **(D, E)** Transwell migration and matrigel invasion assays showed that MSAB reduced migration and invasion cells of IARS2 overexpressed BxPC-3 cells. **(F)** The expression levels of EMT related protein in modified PDAC cell lines were assayed by western blotting. β-Actin was used as a loading control. Data represented the mean ± standard deviation of at least three experiments. ∗*P* < 0.05, ∗∗*P* < 0.01, ∗∗∗*P* < 0.001. IARS2, isoleucyl-tRNA synthetase 2; PDAC, pancreatic ductal adenocarcinoma; EMT, epithelial–mesenchymal transition.

**Figure 8:**
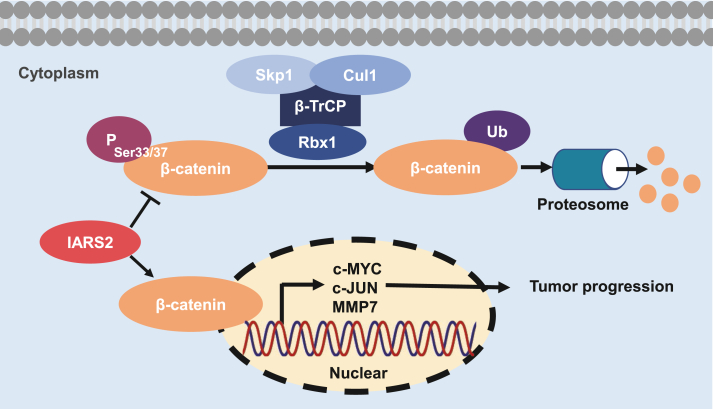
Mechanistic scheme of IARS2 in promoting PDAC progression. IARS2 reduces β-catenin phosphorylation at Ser33/37 site, subsequently attenuating its ubiquitination and inhibiting degradation. Accumulated β-catenin translocates into nuclear and regulates the downstream targets genes, which leads to PDAC progression. IARS2, isoleucyl-tRNA synthetase 2; PDAC, pancreatic ductal adenocarcinoma.

A series of rescue assays were conducted to demonstrate IARS2's contribution to PDAC tumorigenesis via inhibition of β-catenin. MSAB, a compound that binds to β-catenin and promotes its degradation, was added in IARS2 overexpressed BxPc-3 cells. CCK8 assays showed that the difference in proliferation caused by IARS2 was counteracted by MSAB (Fig. 7A). With the increase of MSAB concentration from 0 to 1 μM, the gap of proliferation rate was gradually narrowed (Fig. 7B). Meanwhile, MSAB abolished enhancement of colony formation ability in IARS2 overexpressed cells (Fig. 7C; Fig. S11). Migration and invasion assays showed that MASB reduced migration and invasion abilities promoted by IARS2 (Fig. 7D and E). In addition, MASB treatment reversed the up-regulation of ZEB1, N-cadherin, and Slug induced by the overexpression of IARS2 in BxPC-3 cells (Fig. 7F). Together, these results supported that IARS2 promoted pancreatic tumorigenesis by stabilizing β-catenin (Fig. 8).

## Discussion

With different molecular patterns, PDAC is a heterogeneous disease and responds variably to the same therapy.^2^ Multi-omics analysis identifies a subset of PDAC with WNT-addition, which is sensitive to WNT inhibitors in preclinical study.^3^ Unfortunately, these agents have fallen short of expectations in clinical trials and other crosstalk of WNT signaling remains to be investigated.^24^ IARS2, a protein ligating isoleucine with nucleotide triplets of tRNA, is highly expressed in multiple cancers and promotes tumorigenesis in various organs, but its function in PDAC is unclear. In this study, we explored the role IARS2 played in PDAC and revealed the correlation between IARS2 and WNT signaling pathway.

First, we evaluated the IARS2 transcription level in different cancers using the TCGA database and identified that IARS2 was an oncogene and up-regulated in PDAC. Although IARS2 was increased in pancreatic cancer at grades 3–4, there was no significant alteration among pancreatic cancer at different stages. Considering most patients in the TCGA-PAAD cohort were at stage Ⅰ–Ⅱ, whether the transcription level of IARS2 was irrelevant to the PDAC stage remained to be further confirmed. Next, we explored IARS2 function both *in vitro* and *in vivo* and results demonstrated that IARS2 enhanced PDAC proliferation and metastasis. We found IARS2 promoted EMT because transcription factors such as ZEB1, β-catenin, and Slug were up-regulated by IARS2. EMT is a key event in cancer stemness, metastasis, and chemotherapy resistance,^25^ so we speculate IARS2 promotes PDAC development via EMT, which also partially explains why IARS2 is correlated with poor prognosis.

To figure out other potential mechanisms of IARS2 pro-tumor function, bioinformatic analyses were performed. KEGG enrichment results showed that pathways relating to pancreatic secretion, diabetes, and amino acid metabolism were enriched, indicating that IARS2 might be involved in digestion and glucose regulation. It has been proved that aminoacyl-tRNA synthetases regulated OXPHOS (oxidative phosphorylation) subunits and played a regulatory role in diabetes, which is consistent with our enrichment results.^26^ Meanwhile, type 2 diabetes mellitus is an independent risk factor for pancreatic cancer mortality, thus glucose disorder might be a reason for IARS2 related to poor prognosis.^27^ Next, GSEA analysis was conducted and exhibited the enrichment of cell cycle, apoptosis, p53, and WNT signaling pathway. Previous literature has demonstrated IARS2 was involved in cell cycle, apoptosis, and p53 signaling pathway except WNT signaling.^15^^,^^28^^,^^29^ WNT signaling facilitates PDAC initiation and progression through versatile effects including cellular proliferation, differentiation, and stemness.^30^ Associated with WNT signaling, cancer stem cells have been put forward to be an important part of cancer recurrence. Theoretically, even a single cancer stem cell can reconstitute an entire tumor.^31^ Using a novel stemness index mRNAsi, we found higher expressed IARS2 gained more stem-cell-like features for PDAC. PDAC cancer stem cell markers including CD44, MET, CD133, FUT4, ACVR1, mTOR, and KLF4 were positively related to IARS2 expression.^32^ Additionally, we explored the correlation between IARS2 and tumor immune microenvironment. Immune cell infiltration level was analyzed in 179 PDAC tissues and the results showed a lower infiltration level of CD8 T cells in the high IARS2 expression group. CD8 T cells in PDAC were linked with cytotoxic effects on tumor cells, thus IARS2 might exert immune suppression in the tumor microenvironment of PDAC.^33^ Based on these results, we supposed that IARS2 might affect PDAC proliferation, metastasis, and stemness via the WNT signaling pathway.

As a key factor in the canonical WNT signaling pathway, β-catenin can be rapidly enriched and then imported into the nucleus to regulate gene transcription once WNT signaling pathway is activated.^7^ Therefore, we first evaluated the correlation between CTNNB1 and IARS2 mRNA transcription levels in PDAC cell lines but the results reflected IARS2 was irrelevant to the CTNNB1 at the transcription level. Overexpression or knockdown of IARS2 also failed to interrupt the transcription level of CTNNB1. However, the downstream targets of β-catenin including c-MYC and c-JUN were regulated by IARS2. The protein level of β-catenin was also altered by IARS2 knockdown or overexpression. Therefore, we hypothesized that IARS2 affected β-catenin degradation. Phosphorylated by CK1α (casein kinase 1α) and GSK3 (glycogen synthase kinase 3) on serine and threonine residues (S45/T41/S37/S33) at the N-terminus, β-catenin can be recognized by a ubiquitin ligase complex, which leads to poly-ubiquitination and proteasomal degradation.^34^^,^^35^ CHX assays showed that IARS2 overexpression slowed down the degradation of β-catenin. Meanwhile, phosphorylation at S33/37 was regulated by IARS2 protein expression level and the reduced β-catenin could be elevated by MG-132, which together confirmed our hypothesis. HitPredict database was used to evaluate the protein interaction between IARS2 and β-catenin or degradation protein complex, and the results predicted that IARS2 might bind to GSK3β, which could be the reason that IARS2 could inhibit β-catenin degradation (Table S3). Next, rescue assays were performed and the results showed that the promotion of β-catenin degradation impeded IARS2's effect on PDAC proliferation and metastasis, validating that IARS2 favored PDAC development through β-catenin. Overall, our study provides compelling evidence that IARS2 facilitates PDAC proliferation and metastasis by stabilizing β-catenin. However, some limitations in our study remain to be explored in future research that how IARS2 impacts β-catenin ubiquitination degradation, and more experiments are needed to discover the specific interaction between IARS2 and GSK3β. Additionally, more PDAC tissue and corresponding clinical information of patients should be collected for further validation of the impact that IARS2 exerted on tumor immune microenvironment and metastasis.

In conclusion, we comprehensively characterized IARS2, as an oncogene, could facilitate PDAC proliferation and metastasis by stabilizing β-catenin and activating the WNT/β-catenin pathway. Our results indicate that IARS2 serves as an underlying prognosis marker and a potential therapeutic target for PDAC.

## CRediT authorship contribution statement

Y.J. studied the concept and designed the study. X.H. collected and interpreted the data. Z.W. drafted the manuscript. Q.W. and B.K. revised the manuscript for important intellectual content. L.W. supervised the study and provided administrative and technical support. All authors have made a significant contribution to this study and approved the final manuscript.

## Conflict of interests

The authors declared no conflict of interests.

## Funding

This work was supported by the 10.13039/501100001809National Natural Science Foundation of China (No. 81870385, 81702740).
