# Supplementary material for: Isoleucyl-tRNA synthetase 2 promotes pancreatic ductal adenocarcinoma proliferation and metastasis by stabilizing β-catenin
Source: Genes Dis. 2024 Jul 24;12(3):101382. doi: 10.1016/j.gendis.2024.101382 (PMC11907448; doi:10.1016/j.gendis.2024.101382)
Supplement: Multimedia component 1 [file mmc1.pptx]

## Slide 1
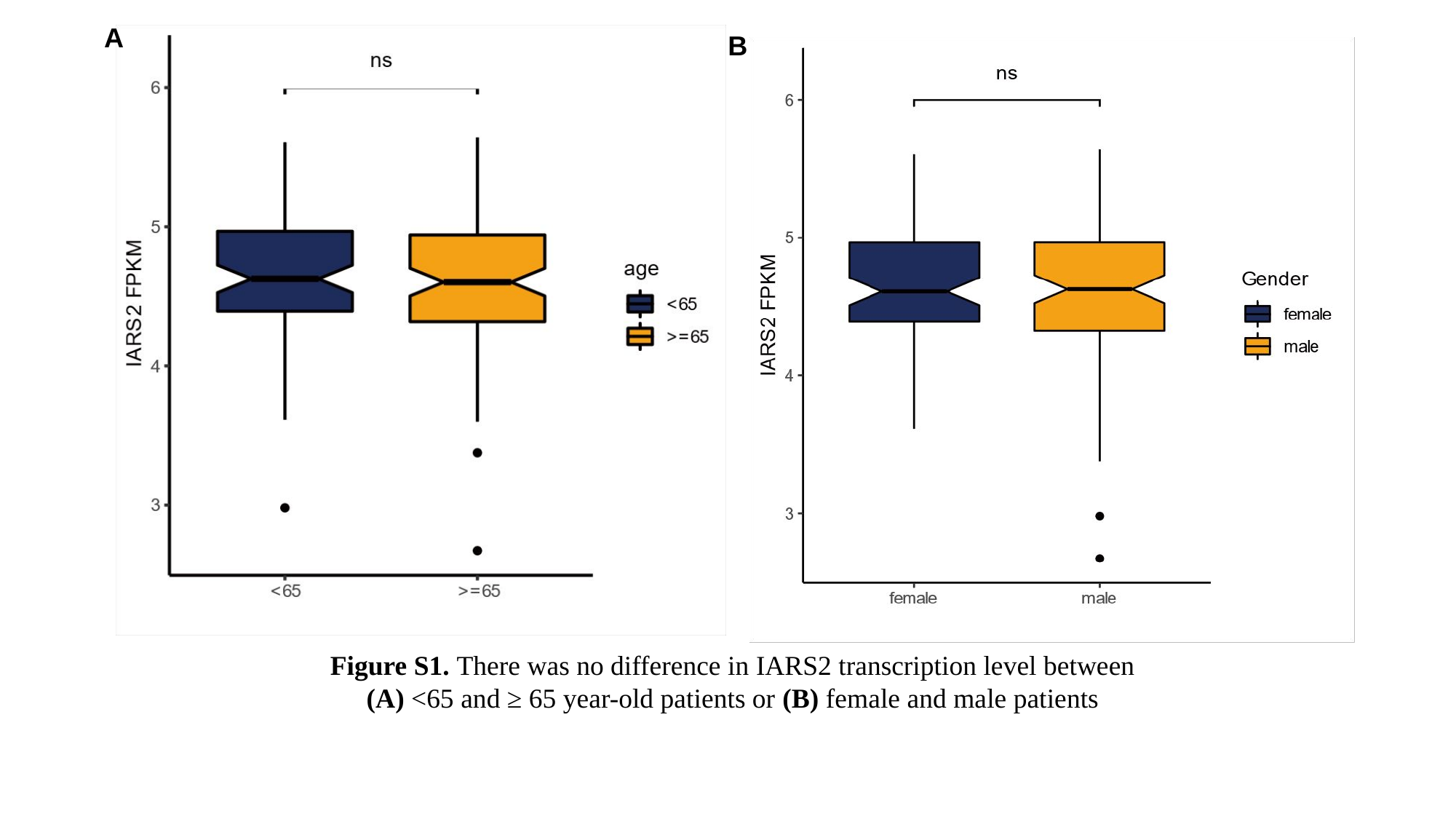

A
B
Figure S1. There was no difference in IARS2 transcription level between (A) <65 and ≥ 65 year-old patients or (B) female and male patients

## Slide 2
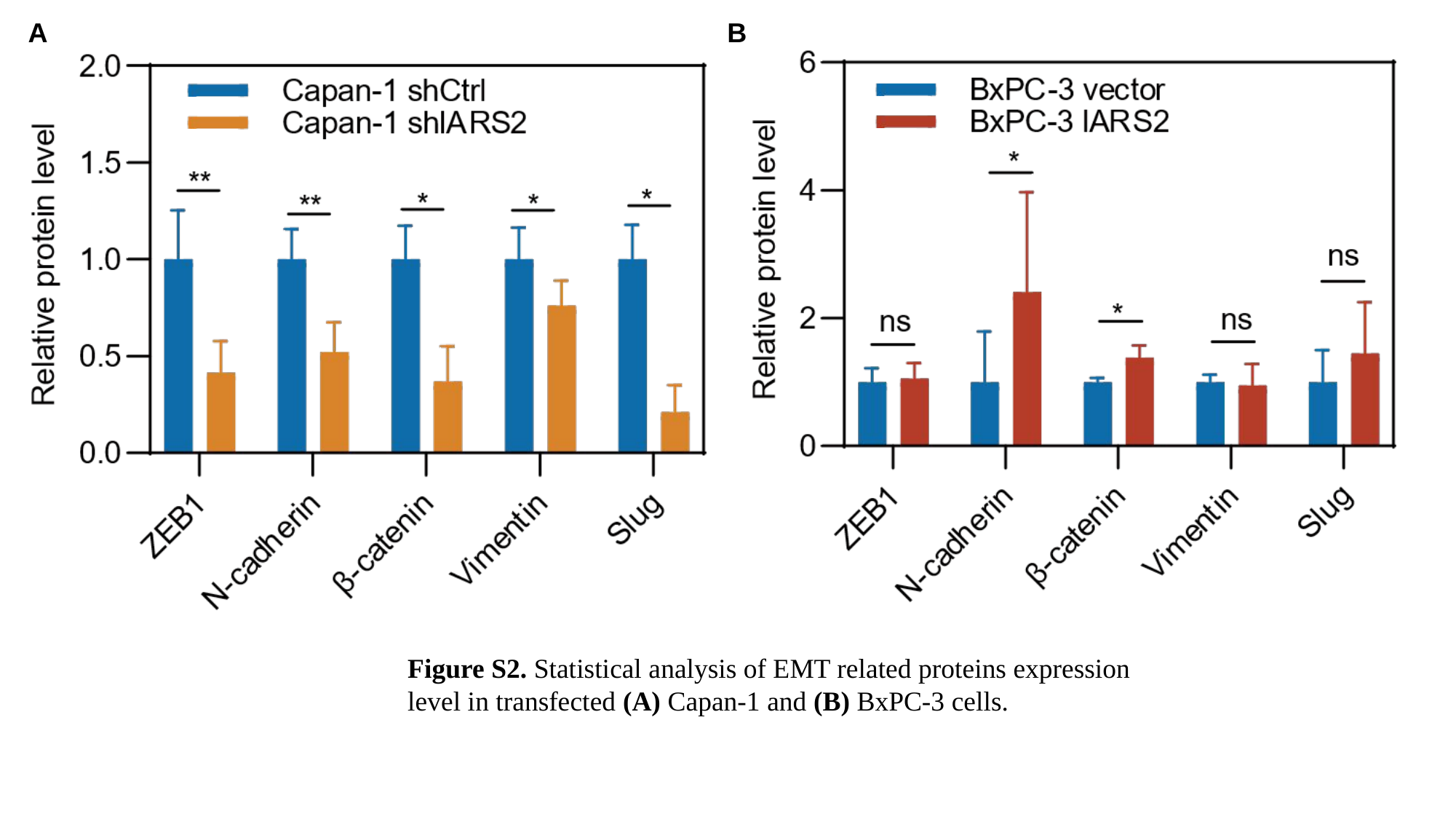

A
B
Figure S2. Statistical analysis of EMT related proteins expression level in transfected (A) Capan-1 and (B) BxPC-3 cells.

## Slide 3
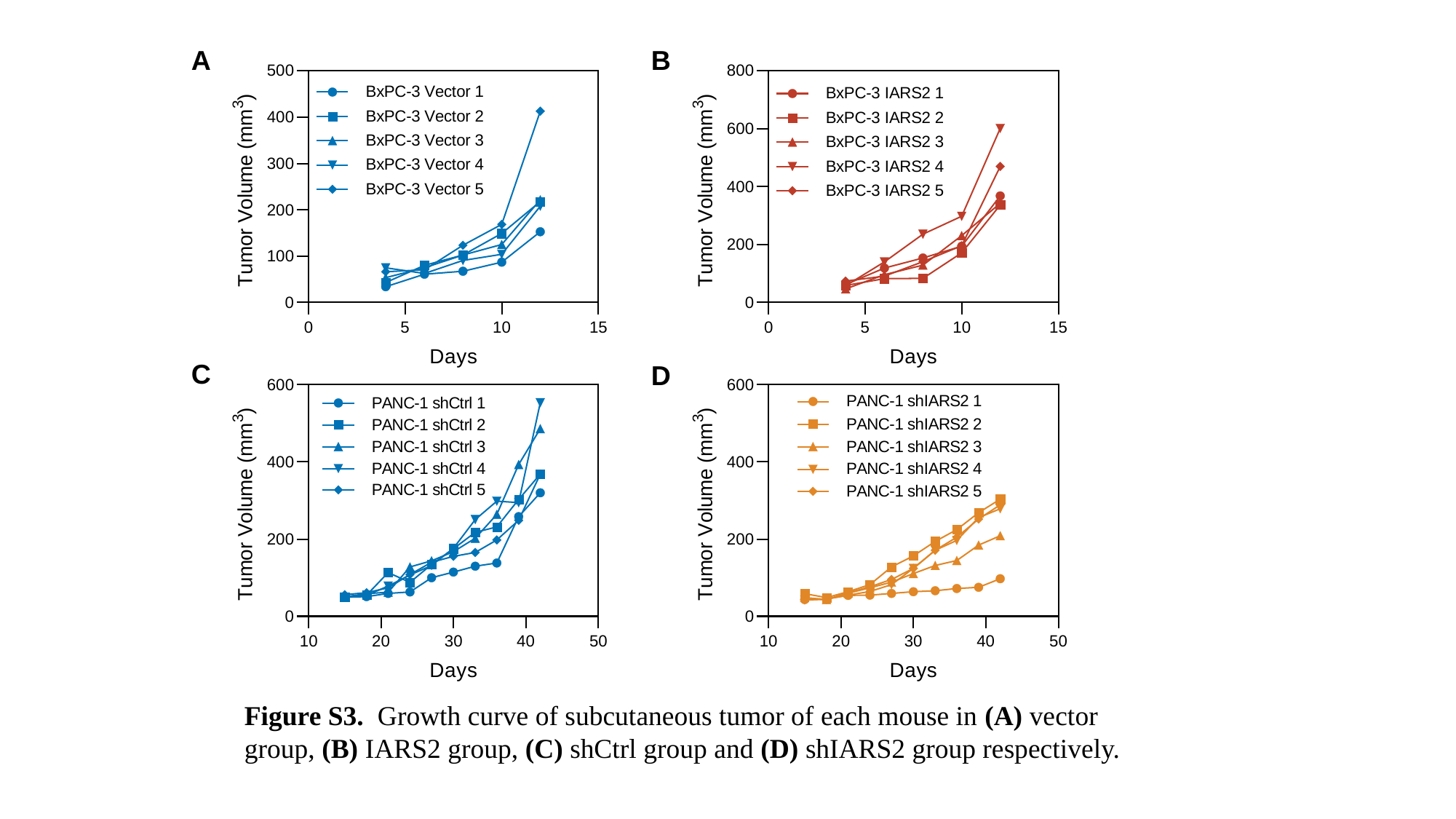

A
B
C
D
Figure S3. Growth curve of subcutaneous tumor of each mouse in (A) vector group, (B) IARS2 group, (C) shCtrl group and (D) shIARS2 group respectively.

## Slide 4
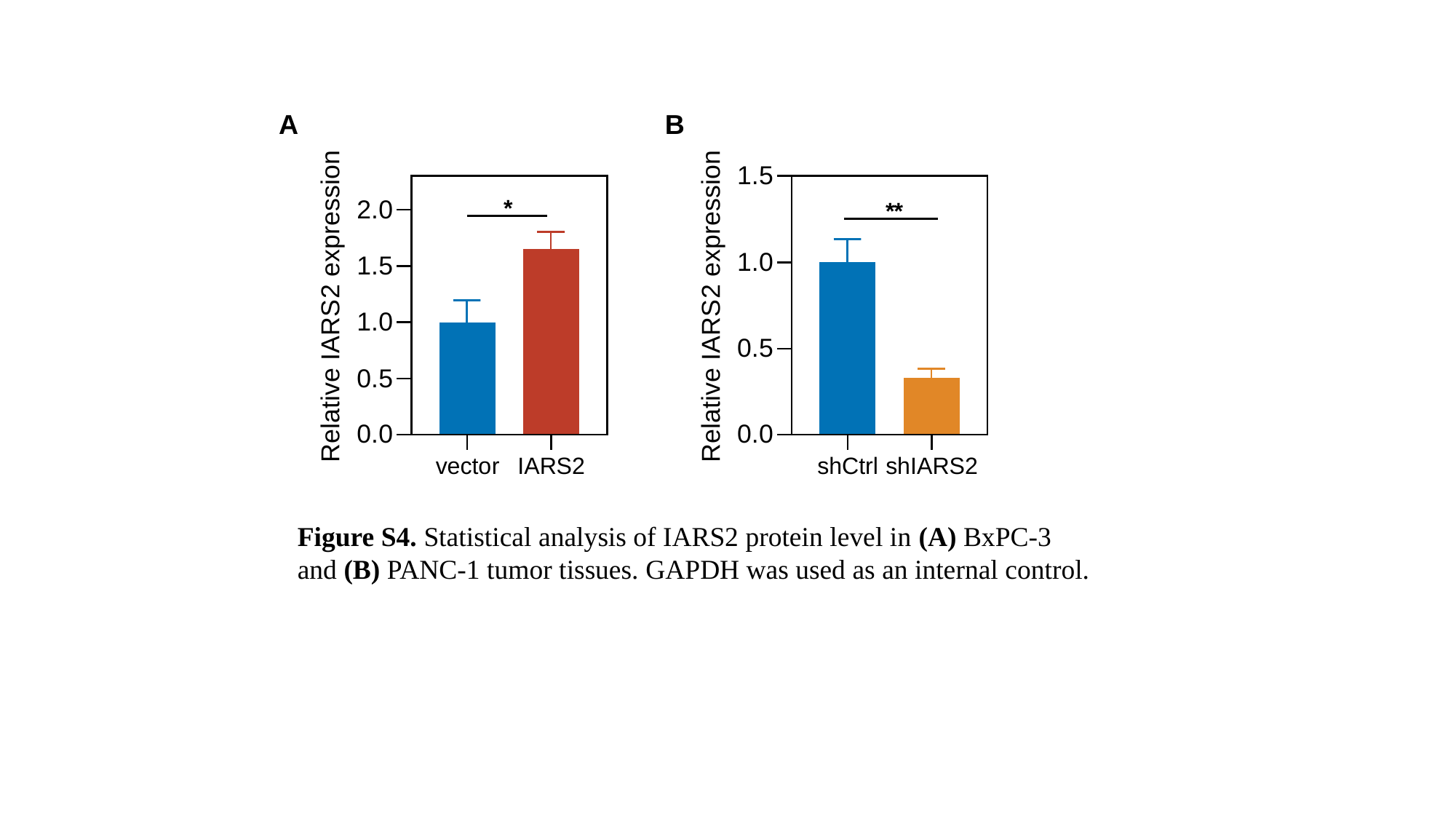

A
B
Figure S4. Statistical analysis of IARS2 protein level in (A) BxPC-3 and (B) PANC-1 tumor tissues. GAPDH was used as an internal control.

## Slide 5
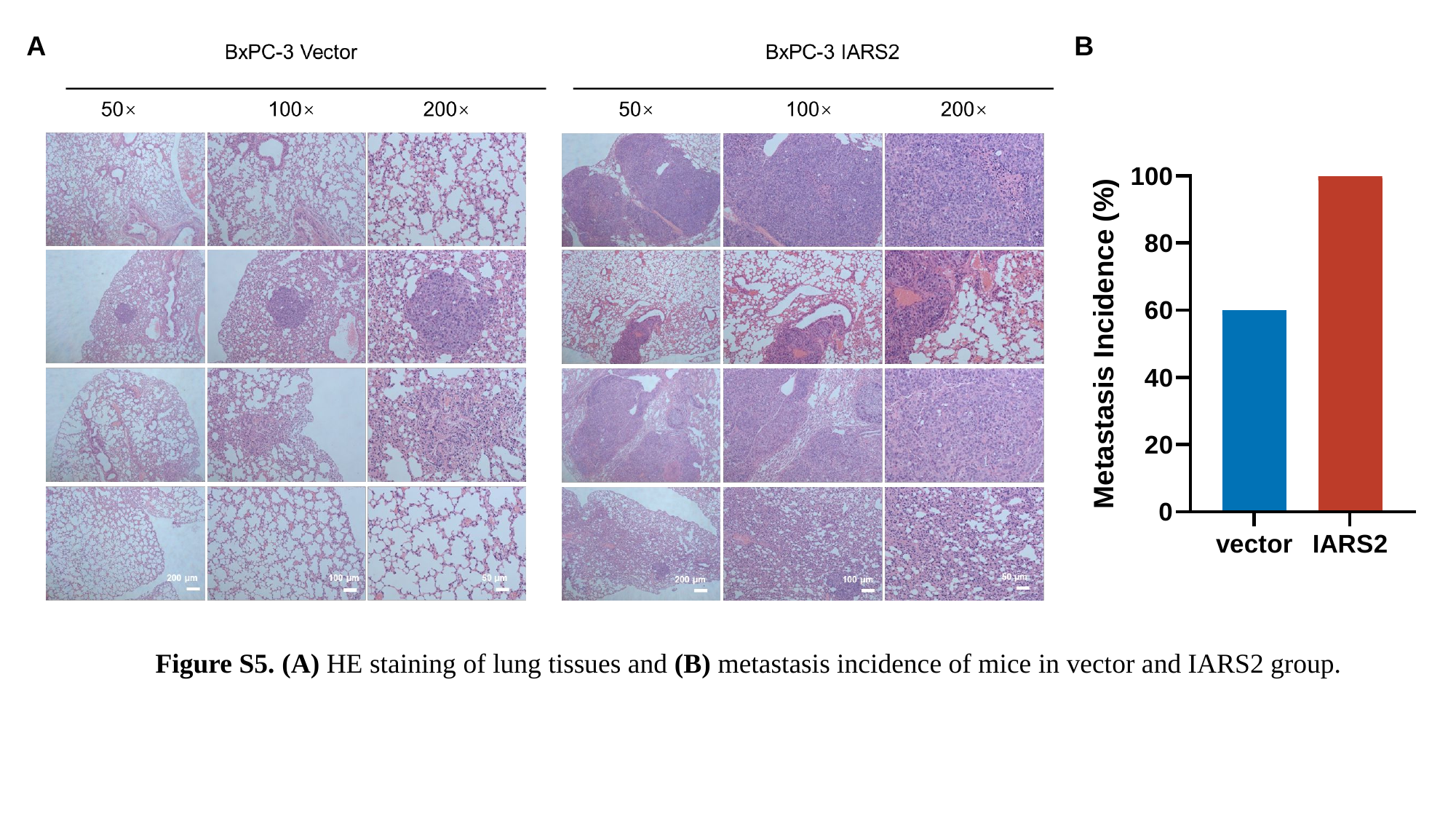

A
B
Figure S5. (A) HE staining of lung tissues and (B) metastasis incidence of mice in vector and IARS2 group.

## Slide 6
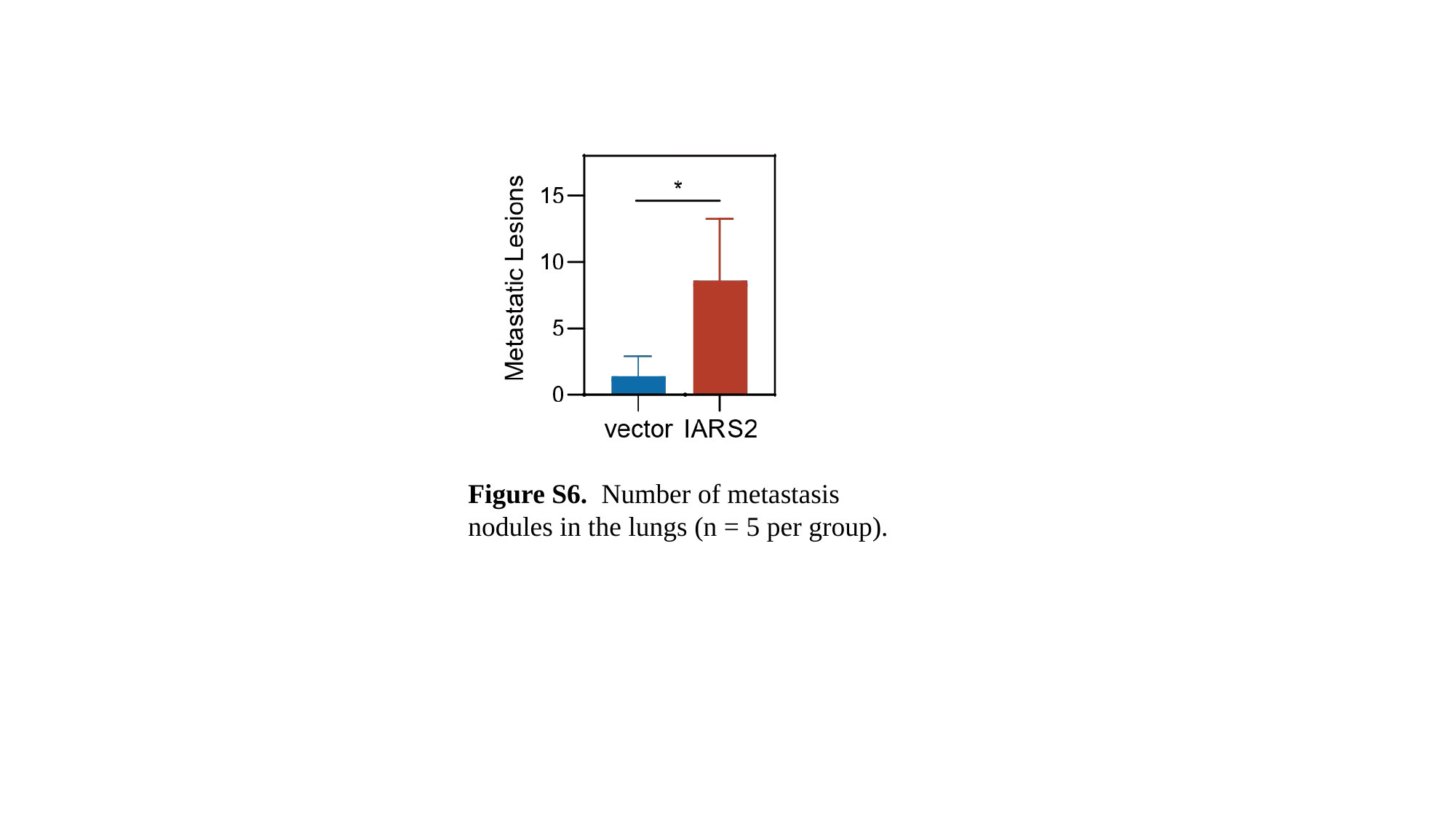

Figure S6. Number of metastasis nodules in the lungs (n = 5 per group).

## Slide 7
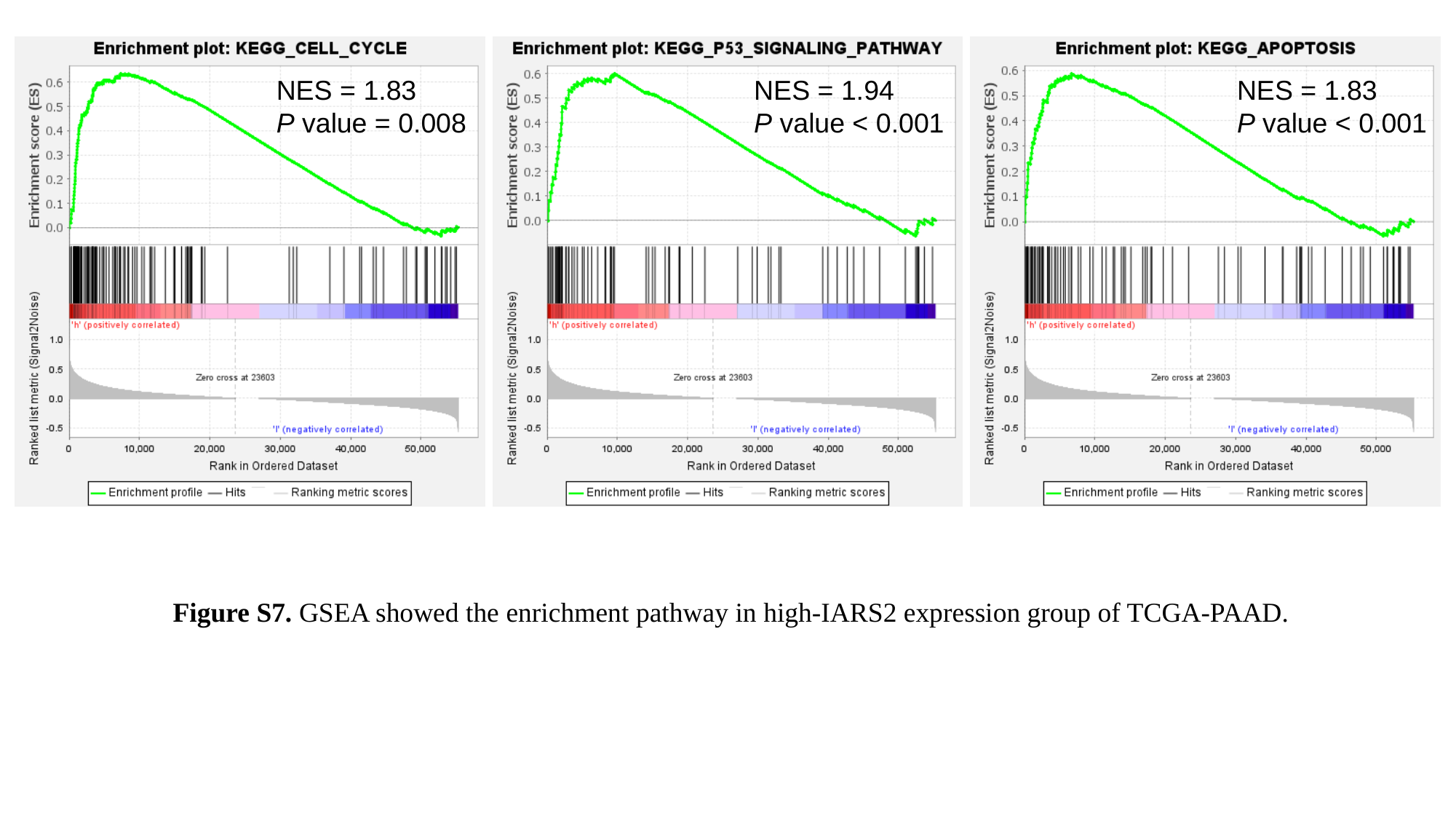

NES = 1.83
P value = 0.008
NES = 1.83
P value < 0.001
NES = 1.94
P value < 0.001
Figure S7. GSEA showed the enrichment pathway in high-IARS2 expression group of TCGA-PAAD.

## Slide 8
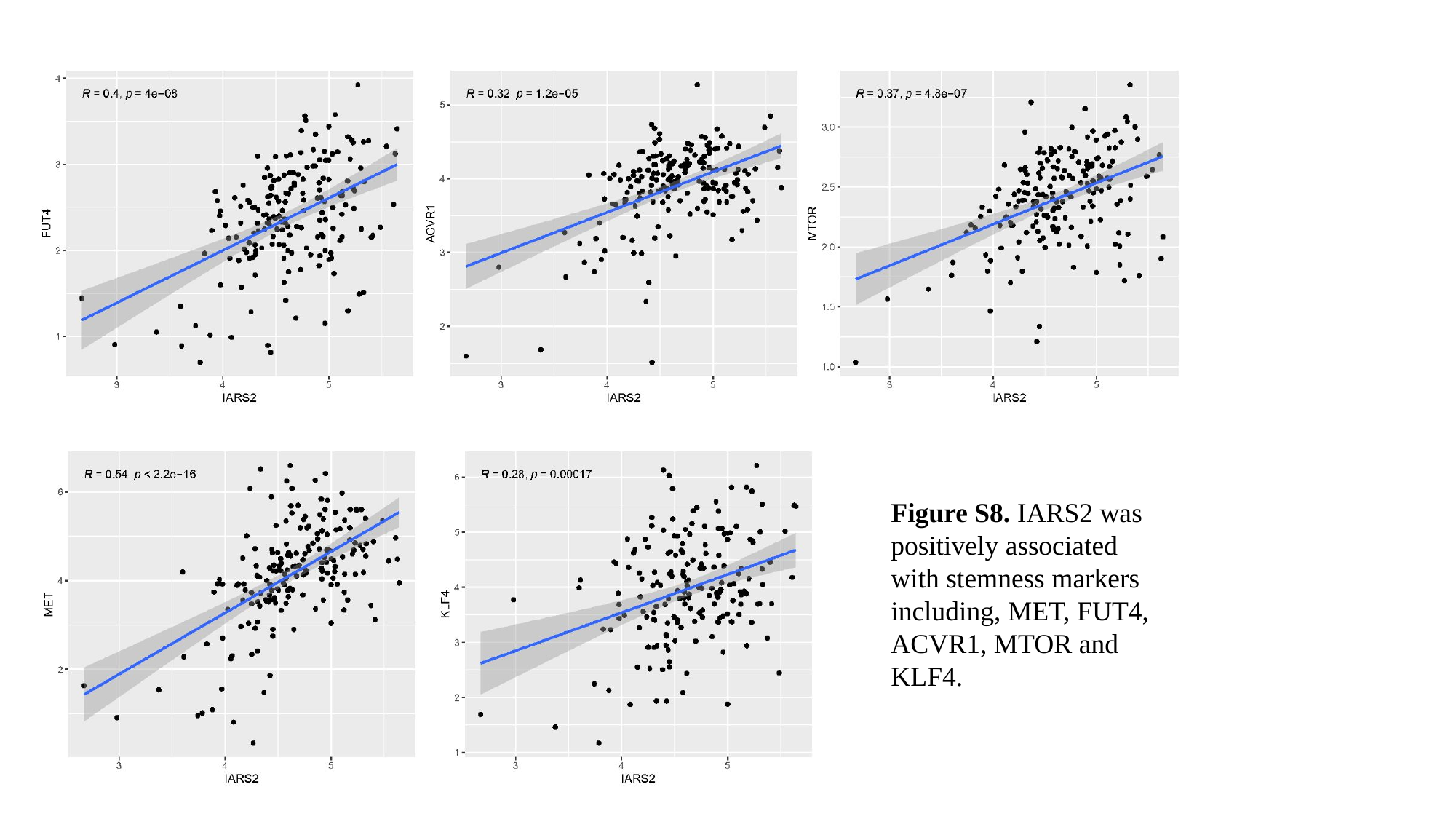

Figure S8. IARS2 was positively associated with stemness markers including, MET, FUT4, ACVR1, MTOR and KLF4.

## Slide 9
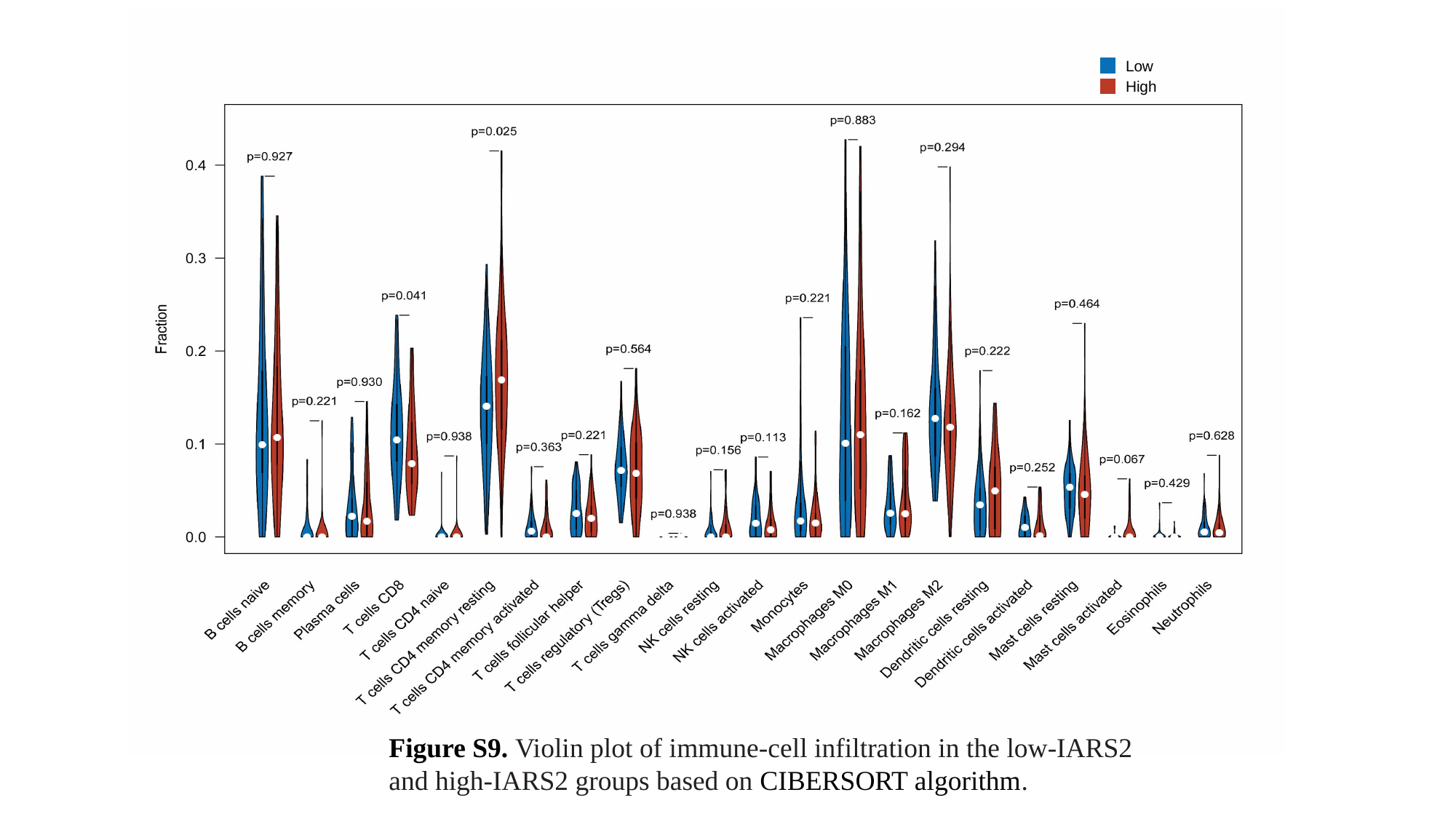

Low
High
Figure S9. Violin plot of immune-cell infiltration in the low-IARS2 and high-IARS2 groups based on CIBERSORT algorithm.

## Slide 10
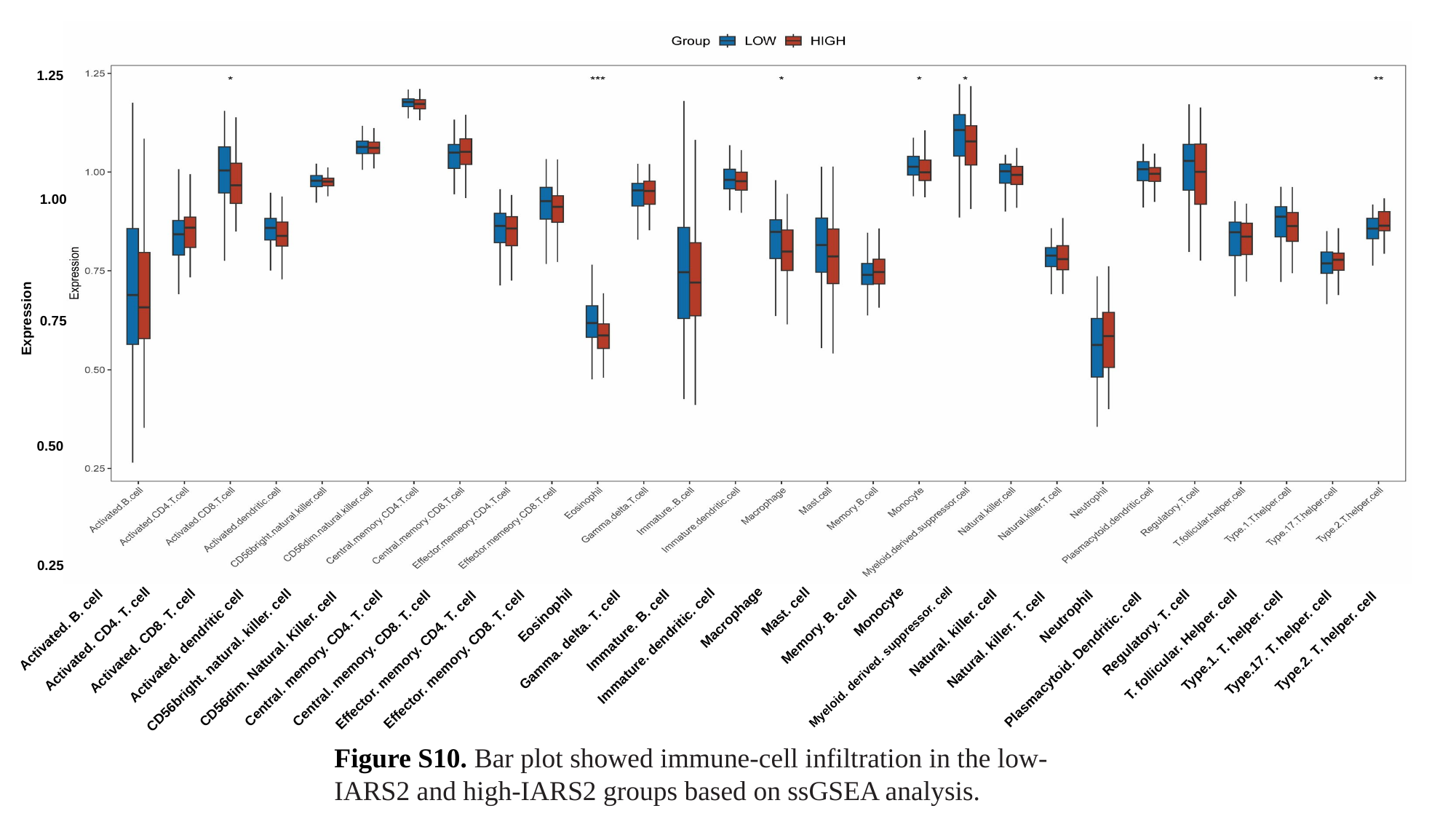

1.25
1.00
Expression
0.75
0.50
0.25
Mast. cell
Monocyte
Eosinophil
Neutrophil
Macrophage
Memory. B. cell
Activated. B. cell
Immature. B. cell
Regulatory. T. cell
Natural. killer. cell
Activated. CD4. T. cell
Natural. killer. T. cell
Gamma. delta. T. cell
Type.1. T. helper. cell
Activated. CD8. T. cell
Type.2. T. helper. cell
Type.17. T. helper. cell
T. follicular. Helper. cell
Activated. dendritic cell
Immature. dendritic. cell
Myeloid. derived. suppressor. cell
Central. memory. CD4. T. cell
Central. memory. CD8. T. cell
CD56dim. Natural. Killer. cell
Plasmacytoid. Dendritic. cell
Effector. memory. CD8. T. cell
CD56bright. natural. killer. cell
Effector. memory. CD4. T. cell
Figure S10. Bar plot showed immune-cell infiltration in the low-IARS2 and high-IARS2 groups based on ssGSEA analysis.

## Slide 11
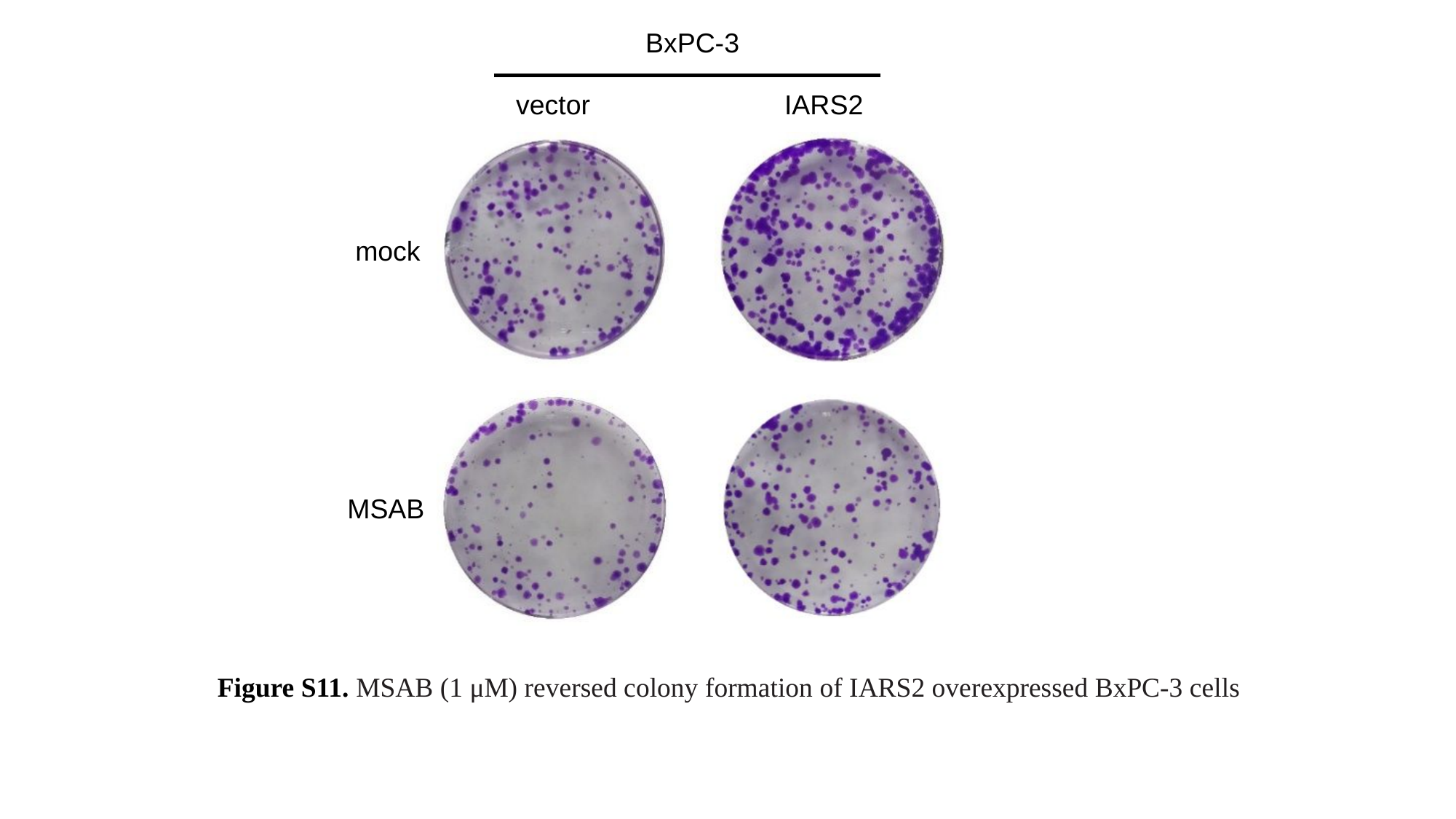

BxPC-3
vector
IARS2
mock
MSAB
Figure S11. MSAB (1 μM) reversed colony formation of IARS2 overexpressed BxPC-3 cells
